# Interleukin-30 Suppresses Not Only CD4^+^ T Cells but Also Regulatory T Cells in Murine Primary Biliary Cholangitis

**DOI:** 10.3390/biomedicines9081031

**Published:** 2021-08-17

**Authors:** Hung-Wen Chen, Chia-I. Lin, Ya-Hui Chuang

**Affiliations:** 1 Department of Clinical Laboratory Sciences and Medical Biotechnology, College of Medicine, National Taiwan University, Taipei 10617, Taiwan; celestechen43@gmail.com (H.-W.C.); wulala08ali@gmail.com (C.-I.L.); 2 Department of Laboratory Medicine, National Taiwan University Hospital, Taipei 10617, Taiwan

**Keywords:** primary biliary cholangitis, autoimmune disease, interleukin-30, immune therapy, CD4^+^ T cells

## Abstract

Primary biliary cholangitis (PBC) is a chronic liver autoimmune disease with augmented T helper (Th) 1 and corresponding cytokine IFN-γ immune responses. Using 2-octynoic acid (2-OA) coupled to OVA (2-OA-OVA)-induced mouse models of autoimmune cholangitis (inducible chemical xenobiotic models of PBC), our previous study demonstrated that overexpression of IFN-γ in the model mice enhanced liver inflammation upon disease initiation, but subsequently led to the suppression of chronic inflammation with an increase in interleukin-30 (IL-30) levels. In this study, we investigated whether IL-30 had an immunosuppressive function and whether it could be part of an immune therapeutic regimen for PBC, by treating model mice with murine IL-30-expressing recombinant adeno-associated virus (AAV-mIL-30). We first defined the effects of AAV-mIL-30 in vivo by administering it to a well-known concanavalin A (ConA)-induced hepatitis model of mice and found that AAV-mIL-30 reduced the numbers of activated CD25^+^CD4^+^ T cells and the levels of serum IFN-γ and IL-12. In autoimmune cholangitis, decreased numbers of activated CD4^+^ T cells and Foxp3^+^ regulatory T cells were noted in the mice treated with AAV-mIL-30 at 3 weeks after the 2-OA-OVA immunization. Treatment with IL-30 did not change the features of autoimmune cholangitis including autoantibodies, cell infiltration, and collagen deposition in the liver at 11 weeks of examination. However, increased levels of cytokines and chemokines were observed. These results suggest that IL-30 suppresses not only CD4^+^ T cells but also regulatory T cells. Additionally, the administration of IL-30 did not suppress liver inflammation in the murine model of PBC.

## 1. Introduction

Primary biliary cholangitis (PBC) is a progressive autoimmune liver disease characterized by the immune-mediated destruction of intrahepatic small bile ducts, leading to decreased bile secretion, fibrosis, and eventual liver failure. The destruction of biliary cells is mediated by liver-infiltrating autoreactive T cells [1,2]. It is commonly accepted that the augmented T helper (Th) 1 response, and corresponding cytokine IFN-γ response, play a significant role in PBC [3,4,5,6,7,8,9,10]. There are a significantly higher number of IFN-γ mRNA-positive cells within the livers of PBC patients than in those of healthy individuals [3,4,5]. Two independent genome-wide association studies have demonstrated that Th1-related IL-12A and IL-12RB2 variants are strongly associated with PBC [6,7]. In experimental mouse models of PBC, there are significantly increased levels of Th1 cytokines, TNF-α and IFN-γ, in the liver [11,12,13]. Moreover, the deletion of IFN-γ in a PBC mouse model reduces inflammatory portal infiltrates associated with the prevention of bile duct damage [10].

In our previous study, by using the chemical xenobiotic 2-octynoic acid (2-OA) coupled to the OVA (2-OA-OVA)-induced PBC mouse model, we demonstrated that the overexpression of IFN-γ enhanced liver inflammation and caused the onset of disease by increasing immune cell infiltrates, upregulating MHC class II expression of antigen-presenting cells (APCs), promoting anti-PDC-E2 antibody production, and activating CD4^+^ and CD8^+^ T and NK cells. However, IFN-γ also protects the portal inflammation by producing IL-30 at the effector phase [8], suggesting that IL-30 may be an immunosuppressive cytokine which can inhibit PBC.

In this study, we investigated whether IL-30 indeed has an immunosuppressive function and whether it can be leveraged in an immune therapeutic regimen for PBC, by treating model mice with murine IL-30-expressing recombinant adeno-associated virus (AAV-mIL-30). Genetically engineered recombinant AAV is desirable for use in gene transfer in vivo, as it has long-term expression, can replicate in a defective state, infect a broad range of cell types, and elicit only a mild immune response compared with the older versions of adenovirus [14]. Moreover, AAV vector serotype DJ used in this study, has a superior transduction efficiency in the liver and is considered an attractive vector for liver-specific gene expression [8,15]. Our results showed that IL-30 suppressed the numbers of not only CD4^+^ T cells but also regulatory T cells (Tregs) and IL-30-induced increased inflammation in the mouse model of PBC.

## 2. Materials and Methods

### 2.1. Experimental Mice

Female C57BL/6 mice aged 7–9 weeks were obtained from the National Laboratory Animal Center, Taipei, Taiwan. Foxp3^GFP^ mice [16], which were kindly provided by Alexander Y. Rudensky (Howard Hughes Medical Institute, and Memorial Sloan Kettering Cancer Center, New York, NY, USA), were maintained in the Animal Center of the College of Medicine, National Taiwan University, Taiwan. All experiments were performed following approval of The Institutional Animal Care and Use Committee (IACUC) of National Taiwan University College of Medicine and College of Public Health.

### 2.2. Preparation of AAV-mIL-30 and AAV-Mock

AAV-mIL-30 was generated by a helper free packaging system (AAV-DJ, Cell Biolabs, San Diego, CA, USA). mIL-30 cDNA was expressed and reversed from IFN-γ-treated mouse macrophages and cloned to the adeno-associated virus vector (pAAV-IRES-GFP). The cloned plasmid was co-transfected with pAAV-DJ and pHelper at a ratio of 1:1:1 into the packaging HEK293 cell line. Viral particles were purified and quantified as previously reported [8,15]. AAV-mock is identical to the above but contains no transgene in the expression cassette.

### 2.3. ConA-Induced Hepatitis Model

Mice were intravenously injected with 10 mg/kg concanavalin A (ConA, Sigma–Aldrich, St. Louis, MO, USA) for the induction of hepatitis. AAV-mIL-30 or AAV-mock (3 × 10^9^ TU/mouse) was administered to mice 5 days before ConA injection. Mice were sacrificed at 4 h after the ConA injection for the analysis of immune cells and liver inflammation.

### 2.4. 2-OA-OVA Immunized Autoimmune Cholangitis Mouse Model

Mice were intraperitoneally immunized with 2-OA-OVA (20 μg) in the presence of complete Freund’s adjuvant (CFA, Sigma–Aldrich, Burlington, MA, USA) and subsequently boosted every 2 weeks with 2-OA-OVA (20 μg in incomplete Freund’s adjuvant (IFA, Sigma–Aldrich, Burlington, MA, USA). Two micrograms of α-galactosylceramide (Funakoshi, Tokyo, Japan) was injected with the first 2-OA-OVA immunization. AAV-mIL-30 or AAV-mock (3 × 10^9^ TU/mouse) was administered to mice at 3 weeks after the first 2-OA-OVA immunization based on our previous study [15]. Mice were sacrificed 5 weeks post-immunization for the analysis of the subsets and function of immune cells, or at 11 weeks post-immunization for liver pathology examination as described below. All experiments were performed a minimum of three times, each with a group size of 3–5 mice.

### 2.5. Quantitative PCR

**Figure 1:**
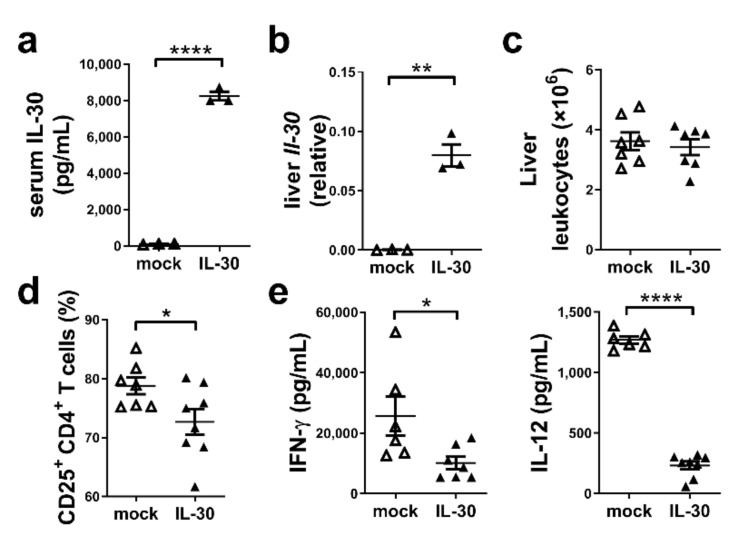
The administration of AAV-mIL-30 reduced the number of CD25^+^CD4^+^ T cells and serum IFN-γ and IL-12 levels in ConA-induced hepatitis mice. Mice were injected with ConA for the induction of hepatitis. AAV-mock or AAV-mIL-30 were administered 5 days before ConA injection. Sera and liver tissues were collected at 4 h after ConA injection. (**a**) Serum levels of IL-30 were detected using ELISA. (**b**) Liver IL-30 mRNA expression was detected using RT-qPCR. Relative quantification was performed with the 2^−ΔCT^ method. (**c**) The number of liver leukocytes was counted. (**d**) The level of CD25 expression on CD4^+^ T cells was analyzed using flow cytometry. (**e**) Serum levels of IFN-γ and IL-12 were detected. Each dot represents an individual mouse. n = 3–7 mice per group. All error bars denote ±SEM. *, *p* < 0.05; **, *p* < 0.01; ****, *p* < 0.0001.

The total RNA from liver specimens was obtained using the TRI^zol^ (Invitrogen Life Technologies, Carlsbad, CA, USA) or Nucleospin^®^ RNA commercial kit (Macherey-Nagel, Düren, Germany) with DNA removal for the detection of IL-30 expression as described in Figure 1b. The cDNA was generated by oligonucleotide priming using High-Capacity cDNA Reverse Transcription Kits (Applied Biosystems, Foster City, CA, USA). Amplification was performed with SYBR Green MasterMix (Thermo Scientific, Waltham, MA, USA) using the 7500 Real-Time PCR System (Applied Biosystems, Waltham, MA, USA). The primer sequences used in PCR were as follows: β-actin forward—CAC AGT GTT GTC TGG TGG TA, reverse—GAC TCA TCG TAC TCC TGC TT; IL-30 forward—GGC CAT GAG GCT GGA TCT C, reverse—AAC ATT TGA ATC CTG CAG CCA. IFN-γ forward—GGC CAT CAG CAA CAA CAT AAG C, reverse—TGG ACC ACT CGG ATG AGC TCA; TNF-α forward—CCC CAA AGG GAT GAG AAG TTC, reverse—TGA GGG TCT GGG CCA TAG AA; Collagen I forward—ACG TCC TGG TGA AGT TGG TC, reverse—CAG GGA AGC CTC TTT CTC CT; Collagen III forward—GTT CTA GAG GAT GGC TGT ACTAAA CAC A, reverse—TTG CCT TGC GTG TTT GAT ATT C. β-actin served as an internal control. Relative quantification was performed using the comparative threshold cycle (CT) method, 2^−ΔCT^.

### 2.6. Liver Mononuclear Cell Quantitation and Cytokine Detection

Mice were sacrificed and their livers were perfused with phosphate-buffered saline containing 0.2% bovine serum albumin and dissociated with the gentleMACS^TM^ Dissociator (Miltenyi Biotec, Auburn, CA, USA). The parenchymal cells were removed as pellets after centrifugation at 50× *g* for 5 min, and the non-parenchymal cells were isolated using 40% and 70% Percoll (GE HealthCare Biosciences, Chicago, IL, Sweden). Subsets of liver mononuclear cells were measured, and functional assays of liver lymphocytes were conducted using flow cytometry. Before staining cells with a previously defined optimal dilution of monoclonal antibodies, the cells were preincubated with anti-CD16/32 (clone 93) to block non-specific FcRγ binding. The following monoclonal Abs were used in this study: anti-CD3 Ab (clone 145-2C11), anti-CD4 Ab (clone GK1.5), anti-CD8a Ab (clone 53–6.7), anti-CD11b Ab (clone M1/70), anti-CD11c Ab (clone N418), anti-CD19 Ab (clone 6D5), anti-CD25 Ab (clone PC61), anti-CD80 Ab (clone 16-10A1), anti-NK1.1 Ab (clone PK136), anti-H-2K^b^ Ab (clone AF6-88.5), anti-I-A^b^ Ab (clone AF6-120.1), and anti-CD45R/B220 Ab (clone RA3-6B2) (Biolegend, San Diego, CA, USA). For IFN-γ detection, liver mononuclear cells were stimulated with phorbol-myristate acetate (PMA, 50 ng/mL, Sigma–Aldrich, Burlington, MA, USA) and ionomycin (1 μg/mL, Sigma–Aldrich, Burlington, MA, USA) for 4 h in the presence of brefeldin A (1 μg/mL) (BD Biosciences). The cells were stained with surface molecules, permeabilized with Cytofix/Cytoperm reagent (BD Biosciences, San Diego, CA, USA) according to the manufacturer’s protocol, and stained with PE-conjugated anti-IFN-γ (clone XMG1.2). The stained cells were measured with a flow cytometer (FACSVerse^TM^, BD Biosciences, San Diego, CA, USA, 2-laser (488 nm and 640 nm), 6 color (4–2) configuration) and analyzed using FlowJo software (Tree Star, Inc., Ashland, OR, USA).

### 2.7. Serum Antimitochondrial Antibodies (AMAs) and Cytokine/Chemokine Detection

Serum titers of IgM and IgG anti-PDC-E2 autoantibodies were measured using ELISA with our standardized recombinant mouse PDC-E2, as described previously [8,15,17,18]. Serum levels of IL-30 were assayed by ELISA (catalog number: DY1834, R&D Systems, Minneapolis, MN, USA). Serum levels of cytokines and chemokines were detected by performing a fluorescent bead-based multiplex immunoassay (LEGENDplex™, Biolegend, San Diego, CA, USA) according to the manufacturer’s protocol and analyzed using the CytoFLEX flow cytometer (Beckman Coulter, Brea, CA, USA).

### 2.8. Histopathology

Portions of liver were excised and immediately fixed with 10% buffered formalin solution for 1 day at room temperature. The paraffin-embedded tissue sections were then cut into 4 and 5 μm slices for H&E staining and Masson’s trichrome staining, respectively.

### 2.9. Statistical Analysis

A two-sided unpaired Student’s t test was used to determine the significant differences between the two groups (Prism 6; Graph-Pad Software, La Jolla, CA, USA). The results are expressed as the mean ± standard error of the mean (SEM).

## 3. Results

### 3.1. Administration of AAV-mIL-30 Reduces the Number of Activated CD25^+^CD4^+^ T Cells and Levels of Serum IFN-γ and IL-12 in ConA-Induced Hepatitis

We first determined the expression and function of our AAV-mIL-30 in vivo using a well-known ConA-induced hepatitis mouse model. Mice received AAV-mIL-30 or AAV-mock 5 days before the ConA injection and were examined at 4 h after the injection. Serum levels of IL-30 were around 8000 pg/mL in AAV-mIL-30-treated mice compared to near 0 pg/mL in AAV-mock-treated mice (Figure 1a). IL-30 was detected mostly in the liver tissue of AAV-mIL-30-treated mice (Figure 1b). Liver leukocyte infiltration was not different between the two experimental groups of mice (Figure 1c). It is recorded that the number of activated CD25^+^CD4^+^ T cells had significantly decreased in AAV-mIL-30-treated mice (*p* < 0.05, Figure 1d and Figure S1). Decreased serum levels of IFN-γ and IL-12 were also noted in AAV-mIL-30-treated, ConA-induced mice (Figure 1e).

### 3.2. Administration of AAV-mIL-30 Decreases the Activation of Liver CD4^+^ T Cells in Autoimmune Cholangitis Mice

**Figure 2:**
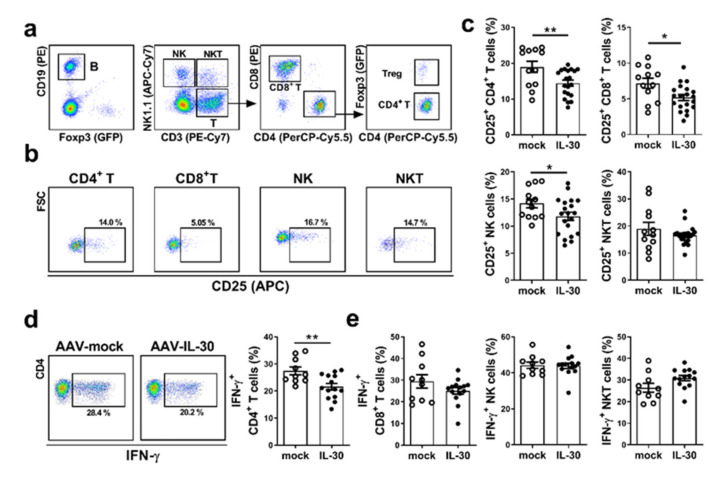
Administration of AAV-mIL-30 decreased the activation of CD4^+^ T cells in 2-OA-OVA-induced autoimmune cholangitis mice. Foxp3^GFP^ mice were injected with AAV-mIL-30 or AAV-mock at 3 weeks after the first 2-OA-OVA immunization and sacrificed at Week 5. Liver leukocytes were isolated. (**a**) Representative flow plots show gating strategies of different subsets of lymphocytes. (**b**) Representative flow plots show CD25 expression on CD4^+^ and CD8^+^ T cells, NK cells, and NKT cells. (**c**) The levels of CD25 expression on CD4^+^ and CD8^+^ T cells, NK cells, and NKT cells were analyzed using flow cytometry. (**d**,**e**) Liver leukocytes were stimulated with phorbol-myristate acetate (PMA) and ionomycin for 4 h, and IFN-γ production in cells was analyzed using intracellular staining and flow cytometry. (**d**) Representative flow cytometry analysis and graphical summary of the level of IFN-γ secretion in liver CD4^+^ T cells. (**e**) The levels of IFN-γ secretion in liver CD8^+^ T, NK, and NKT cells. Each dot represents an individual mouse. n = 12–20 mice per group. All error bars denote ±SEM. *, *p* < 0.05; **, *p* < 0.01.

We then investigated whether AAV-mIL-30 had immunosuppressive function in PBC. Foxp3^GFP^ mice were immunized with 2-OA-OVA for the induction of autoimmune cholangitis. AAV-mIL-30 or AAV-mock was administered at 3 weeks after the 2-OA-OVA immunization. The activation of immune cells was analyzed at 5 weeks after the 2-OA-OVA immunization. As shown in Figure 2c, the levels of activated CD4^+^ T, CD8^+^ T, and NK cells had markedly decreased in the AAV-mIL-30-treated group. Additionally, IFN-γ secretion in the CD4^+^ T cells had also decreased in this group (Figure 2d and Figure S2). However, there were no differences in IFN-γ secretion in the CD8^+^ T, NK, and NKT cells (Figure 2e). Collectively, these results suggest that IL-30 decreases the activation of CD4^+^ T cells in 2-OA-OVA-immunized mice.

### 3.3. Administration of AAV-mIL-30 Decreases the Number of Liver Tregs in Autoimmune Cholangitis Mice

**Figure 3:**
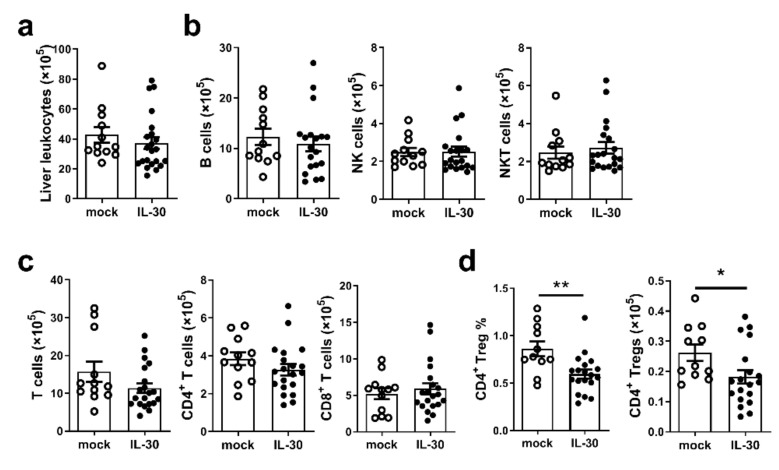
Administration of AAV-mIL-30 decreased the numbers of Foxp3-Tregs in 2-OA-OVA-induced autoimmune cholangitis mice. Foxp3^GFP^ mice were injected with AAV-mIL-30 or AAV-mock at 3 weeks after the first 2-OA-OVA immunization and sacrificed at Week 5. (**a**) The numbers of leukocytes in the liver were measured. (**b**) The numbers of B, NK, and NKT cells in the liver were measured. (**c**) The numbers of CD4^+^ and CD8^+^ T cells in the liver were measured. (**d**) The percentages and numbers of CD4^+^ Foxp3-Tregs in the liver were measured. Each dot represents an individual mouse. n = 12–20 mice per group. All error bars denote ±SEM. *, *p* < 0.05; **, *p* < 0.01.

We then examined the phenotypes of liver-infiltrating lymphocytes in the mice 5 weeks after the 2-OA-OVA immunization. We found that the number of total leukocytes in the liver was not different in mice treated with AAV-mock from those treated with AAV-mIL-30 (Figure 3a). Lymphocyte subsets including B, NK, NKT, CD4^+^ T and CD8^+^ T cells were also not different between the two groups (Figure 3b,c). The expressions of IFN-γ and TNF-α in the liver were not different between the two groups (Figure S3a). Serum levels of IFN-γ, TNF-α, IL-6, CCL-2, CXCL1, CCL5, and IL-1β were not different between the two groups (Figure S3b). However, the percentage and number of CD4^+^ Tregs were significantly decreased in AAV-mIL-30-treated mice (*p* < 0.05, Figure 3d). These results suggest that IL-30 decreases the number of Tregs in the 2-OA-OVA-immunized mice.

### 3.4. IL-30 Does Not Affect Antigen-Presenting Cells in Autoimmune Cholangitis Mice

**Figure 4:**
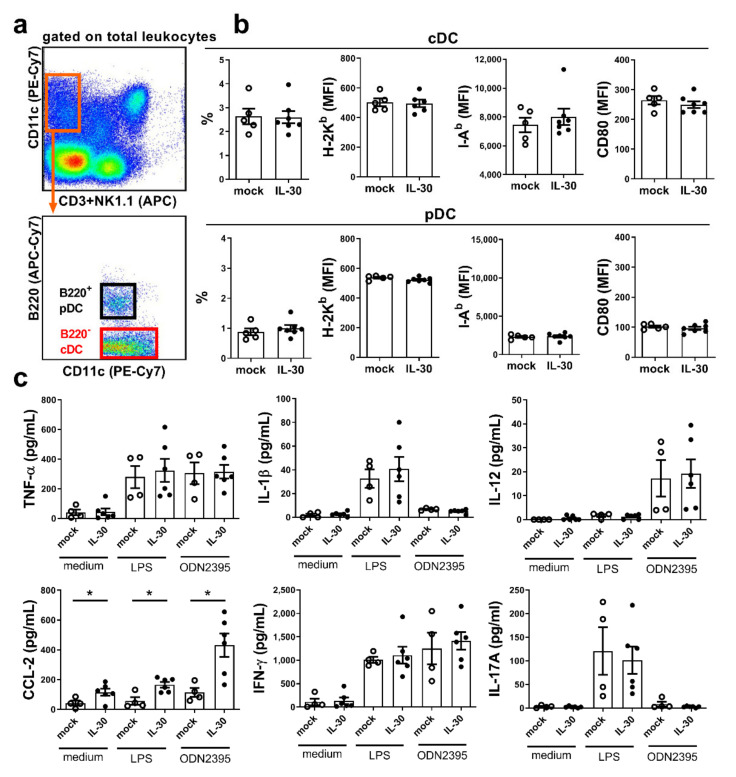
IL-30 did not affect antigen-presenting cells in 2-OA-OVA-induced autoimmune cholangitis mice. Mice were injected with AAV-mIL-30 or AAV-mock at 3 weeks after the first 2-OA-OVA immunization and sacrificed at Week 5. (**a**) Representative flow plots show gating strategies of conventional dendritic cells (cDCs) and plasmacytoid dendritic cells (pDCs). (**b**) The percentage and H-2K^b^, I-A^b^, and CD80 expression of cDCs and pDCs were detected using flow cytometry. cDCs were identified as CD3^−^NK1.1^−^ CD11c^+^B220^−^ cells, whereas pDCs were identified as CD3^−^NK1.1^−^CD11c^+^B220^+^ cells. (**c**) Liver leukocytes were isolated, stimulated with LPS or ODN2395, and their cytokine/chemokine production was detected by performing a fluorescent bead-based multiplex immunoassay. Each dot represents an individual mouse. n = 4–6 mice per group. All error bars denote ±SEM. *, *p* < 0.05.

To determine whether IL-30 affects T cells via APCs, we analyzed the population and the antigen-presenting function of dendritic cells in mice at 5 weeks after the 2-OA-OVA immunization. As shown in Figure 4, the levels of functional molecules, including MHC class I, MHC class II, and CD80, of conventional dendritic cells and plasmacytoid dendritic cells, were not different between the two groups of mice (Figure 4a,b). Liver leukocytes from the AAV-mIL-30-treated and from the AAV-mock-treated mice were isolated and stimulated with lipopolysaccharide (LPS) (TLR4 agonist) or ODN2395 (TLR9 agonist). Levels of cytokines/chemokines produced in the culture supernatants were measured. The production of TNF-α, IL-1β, IL-12, CCL-2, IFN-γ, and IL-17A was found to increase when liver leukocytes were stimulated with LPS or ODN2395. The CCL-2 production of unstimulated and stimulated leukocytes in the AAV-mIL-30-treated mice was increased, compared to that in the mock controls. The production of TNF-α, IL-1β, IL-12, IFN-γ, and IL-17A was not different between the two groups (Figure 4c).

### 3.5. Administration of AAV-mIL-30 Does Not Alleviate the Disease but Increases Cytokine Production in Autoimmune Cholangitis Mice

**Figure 5:**
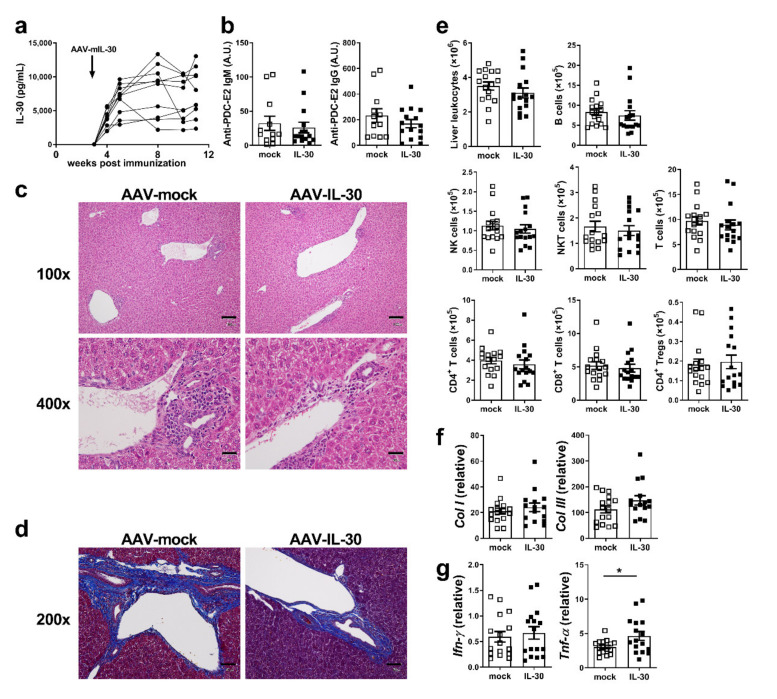
Administration of AAV-mIL-30 did not alleviate the disease. Mice were injected with AAV-mIL-30 or AAV-mock at 3 weeks after the first 2-OA-OVA immunization and sacrificed at Week 11. (**a**) Serum levels of IL-30 in AAV-mIL-30-injected mice. IL-30 was undetectable in mice treated with AAV-mock. (**b**) Serum levels of anti-PDC-E2 IgM and IgG were measured using ELISA. A.U. arbitrary unit. (**c**,**d**) Representative pictures of liver tissue stained with (**c**) hematoxylin and eosin (H&E) (scale bar, 100×, 100 μm; 400×, 300 µm) and (**d**) Masson’s trichrome (scale bar, 50 µm). (**e**) Liver cell infiltrates were counted. (**f**) The expression of collagen I and collagen III mRNA in the liver was detected using RT-qPCR. (**g**) Liver IFN-γ and TNF-α mRNA expression was detected using RT-qPCR. Relative quantification was performed by 2^−ΔCT^ method and multiplied by 10,000. Each dot represents an individual mouse. *n* = 15–17 mice per group. All error bars denote ±SEM. *, *p* < 0.05.

**Figure 6:**
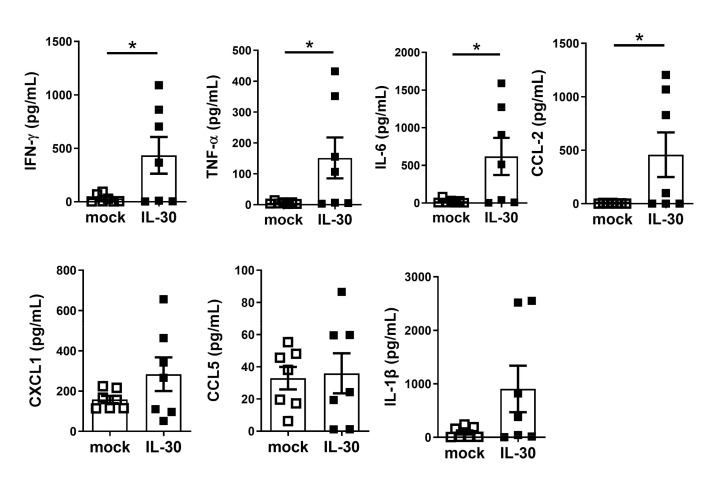
Administration of AAV-mIL-30 induced an increase in the levels of various cytokines and chemokines. Mice were injected with AAV-mIL-30 or AAV-mock at 3 weeks after the 2-OA-OVA immunization, and the serum levels of cytokines and chemokines were measured at Week 11 by performing fluorescent bead-based multiplex immunoassay. Each dot represents an individual mouse. n = 15–17 mice per group. All error bars denote ±SEM. *, *p* < 0.05.

We further examined the therapeutic effect of IL-30 in PBC. Mice were immunized with 2-OA-OVA to induce autoimmune cholangitis. AAV-mIL-30 or AAV-mock was administered at 3 weeks after the 2-OA-OVA immunization. The features of autoimmune cholangitis in the 2-OA-OVA-immunized mice were analyzed at 11 weeks after immunization. IL-30 was highly expressed in the serum of AAV-mIL-30-injected mice up to at least 8 weeks. By contrast, the serum levels of IL-30 were undetectable in the control group (Figure 5a). There were no differences in serum anti-PDC-E2 IgM and IgG levels between the two groups (Figure 5b). The liver histopathology was examined using the H&E stain and the Masson’s trichrome stain. The portal inflammation and collagen deposition between the AAV-mock and AAV-mIL-30-treated groups were not different (Figure 5c,d). There were also no differences in the number of total liver-infiltrating leukocytes and different subsets between the two groups (Figure 5e). The expression of collagen I and III and IFN-γ was not different between the two groups (Figure 5f,g). However, the expression of TNF-α in the liver tissue was significantly increased in AAV-mIL-30-treated mice (*p* < 0.05, Figure 5g). Furthermore, serum levels of IFN-γ, TNF-α, IL-6, CCL-2, CXCL1, CCL5, and IL-1β were markedly increased in the AAV-mIL-30-treated group (Figure 6).

## 4. Discussion

IL-30, a subunit p28 of IL-27, has been detected in macrophages and dendritic cells. IL-30 can form distinct complexes with various partnering subunits such as EBi3, cytokine-like factor 1 (CLF1), IL-12p40, and sIL-6Ra, and perform overlapping and also discrete immunomodulatory functions [19]. In this study, we investigated whether IL-30 had an immunosuppressive function in PBC. Our results showed decreased levels of activated CD4^+^ T cells in mice treated with AAV-mIL-30 at 3 weeks after the 2-OA-OVA immunization. The number of Foxp3^+^ Tregs was also decreased in IL-30-treated mice. Treatment with IL-30 did not change the features of autoimmune cholangitis including autoantibodies, cell infiltration, and collagen deposition at 11 weeks of examination. However, increased levels of cytokines and chemokines were noted. These results suggest that IL-30 not only suppresses the number of CD4^+^ T cells but also the number of Tregs and consequently leads to increased inflammation in murine PBC.

In this study, we found that the administration of AAV-mIL-30 decreased the activation and IFN-γ production of CD4^+^ T cells in autoimmune cholangitis. This result is similar to that of previous studies where the administration of IL-30-encoding plasmids decreases the intrinsic ability of CD4^+^ T cells to produce IFN-γ and where IL-30 conditional knockout mice produce extremely high levels of IFN-γ when injected with ConA [20,21]. However, the immunosuppressive function of IL-30 has been reported in autoimmune diseases such as experimental autoimmune encephalomyelitis and experimental autoimmune uveitis [22,23], though not in liver autoimmune diseases. As per the current study findings, the administration of AAV-mIL-30 did not alleviate autoimmune cholangitis features probably owing to the reduction in the number of Tregs.

Tregs represent a functionally distinct subset of mature T cells, which have a critical role in the development and maintenance of immune tolerance. Tregs can control immune responses directly by suppressing the effector functions of various cell types including CD4 and CD8 T cells, B cells, NK cells, or NKT cells, and also indirectly through changing the function of APCs [24]. In PBC patients, Foxp3^+^ Tregs can be identified in the lymphoid infiltrates localized to the portal tracts. However, significantly lower production of the circulating CD4^+^ CD25^high^ Tregs has been observed in patients and their first-degree family members, compared to that of healthy individuals [25]. By performing the adoptive transfer of cells from the dominant negative TGF-β receptor II PBC model mice to naïve normal mice, previous studies demonstrated that PBC required defects in both effector T cells and Tregs, and that an intrinsic T cell effector defect was not sufficient to mediate autoimmune disease when Tregs were intact [26]. Additionally, the adoptive transfer of Tregs from normal mice significantly reduces the pathology of PBC in dominant negative TGF-β receptor II mice [27]. Although limited independent research is available, these results suggest that Treg dysfunction may be critical in PBC. A previous study established the inhibitory effect of IL-30 on Tregs in terms of reducing the capacity of CD4^+^ T cells to express IL-10 under stimulation with TGF-β plus IL-6 in vitro [22].

The administration of IL-30 was found to reduce the number of activated CD4^+^ T cells and Tregs in autoimmune cholangitis mice compared to that of controls, but there were no changes in the numbers and functions of APCs. Another earlier in vitro study showed that CD4^+^ T cells isolated from mice stimulated with recombinant IL-30-induced the phosphorylation of STAT3 in T cells [28], suggesting that T cells could directly respond to the IL-30. However, we found that the AAV-mIL-30 -administered, ConA-induced mice models with liver injuries had lower IL-12 levels than the AAV-mock control group of mice. This suggests that IL-30 may affect APCs such as macrophages and dendritic cells, and that IL-30 reduces the production of the cytokine IL-6 and prevents macrophage-mediated acute liver injury [29].

In this study, we found increased serum levels of CCL2 in the AAV-mIL-30-treated group. In addition, leukocytes isolated from the livers of AAV-mIL-30-treated mice secreted more CCL-2 than those in the mock controls. CCL2, also known as monocyte chemoattractant protein-1, is a chemokine which recruits macrophages, dendritic cells, and T cells to the sites of inflammation produced by tissue injury. Upon exposure to inflammatory stimuli, such as LPS, TNF-α and IFN-γ, CCL2 is expressed by a variety of cells, such as endothelial cells, epithelial cells, macrophages, dendritic cells and T cells [30]. In PBC, the expression of CCL2 is also found in biliary epithelial cells in inflamed and damaged small bile ducts [31], which may promote the infiltration of corresponding CCR2-expressing cells and further aggravate inflammation in the bile duct lesion in PBC [32].

## 5. Conclusions

**Figure 7:**
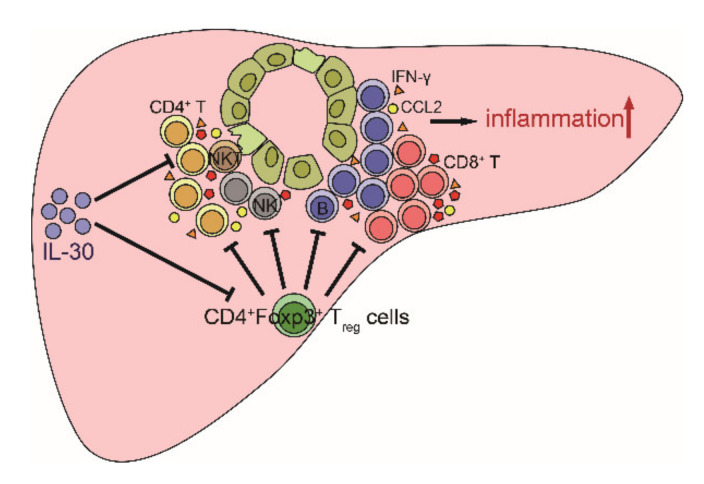
Schematic summary of this study. Infiltrating immune cells such as CD4^+^ T, CD8^+^, NK, NKT, and B cells mediate the destruction of intrahepatic small bile ducts and lead to liver inflammation and fibrosis in PBC. Tregs can suppress various immune cells. IL-30 suppresses the number of infiltrating activated CD4^+^ T cells. However, IL-30 also decreases the number of immunosuppressive Tregs. Hence, the administration of IL-30 did not suppress, but slightly enhanced, liver inflammation in the murine model of PBC.

In conclusion, IL-30 has an immunosuppressive effect on effector CD4^+^ T cells in autoimmune cholangitis. However, the administration of IL-30 may not suppress autoimmune cholangitis based on its ability to suppress the number of Tregs (Figure 7).

## Supplementary Materials

The following are available online at https://www.mdpi.com/article/10.3390/biomedicines9081031/s1, Figure S1: Representative flow plots show gating strategies of CD25 expression on CD4+ T cells in Figure 1d, Figure S2: Representative flow plots show gating strategies of IFN-γ expression in liver CD4^+^ T, CD8^+^ T, NK, and NKT cells in Figure 2d,e, Figure S3: Administration of AAV-mIL-30 did not alter cytokines and chemokines in 2-OA-OVA-induced autoimmune cholangitis mice.
