# Supplementary material for: Interleukin-30 Suppresses Not Only CD4+ T Cells but Also Regulatory T Cells in Murine Primary Biliary Cholangitis
Source: Biomedicines. 2021 Aug 17;9(8):1031. doi: 10.3390/biomedicines9081031 (PMC8392158; doi:10.3390/biomedicines9081031)
Supplement: Supplementary file 1 [file biomedicines-09-01031-s001.zip › biomedicines-1302797-supplementary.pdf]

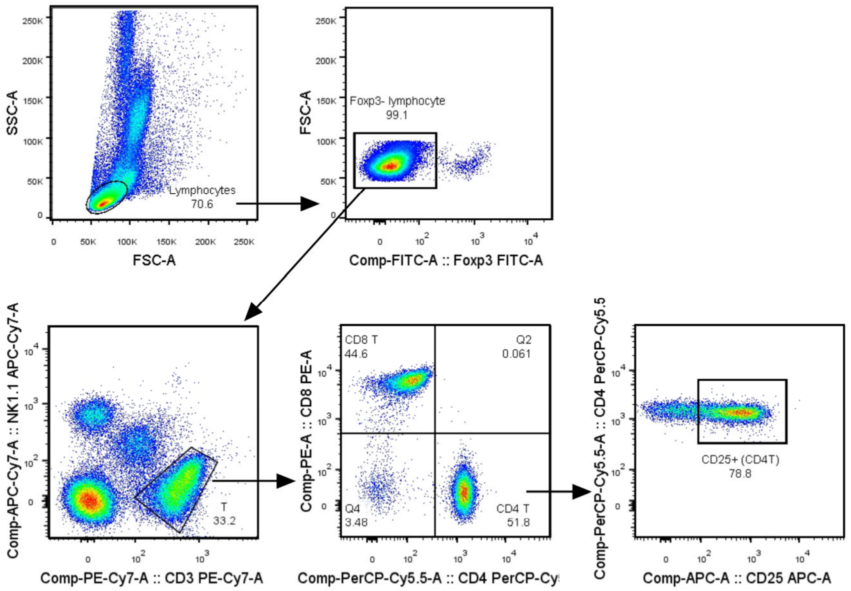

Figure S1. Representative flow plots show gating strategies of CD25 expression on $CD4^+$ T cells in Figure 1d.

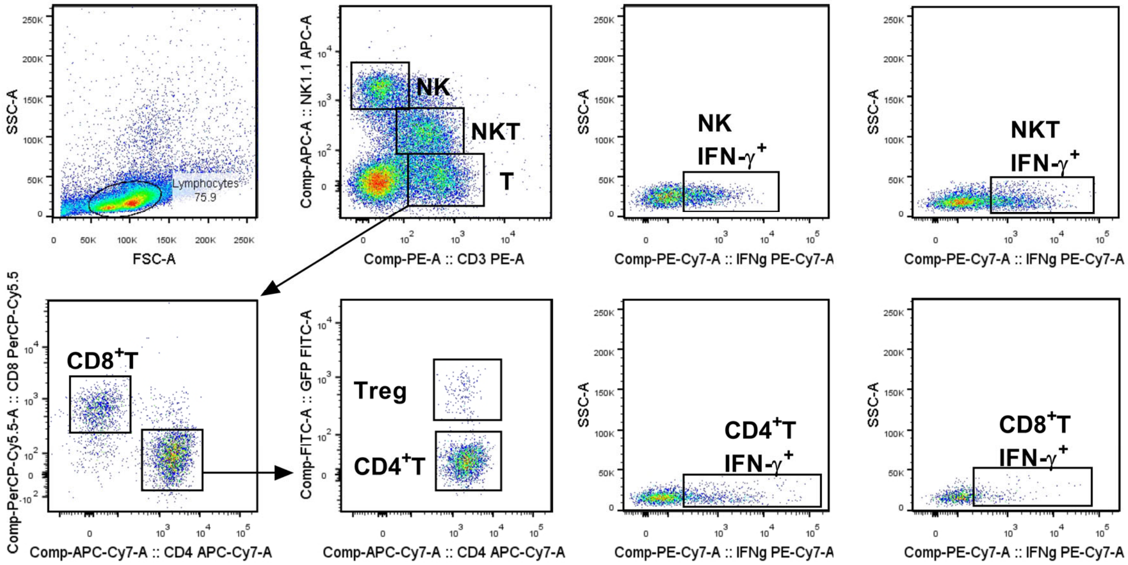

Figure S2. Representative flow plots show gating strategies of IFN-γ expression in liver $CD4^+$ T, $CD8^+$ T, NK, and NKT cells in Figure 2d and 2e.

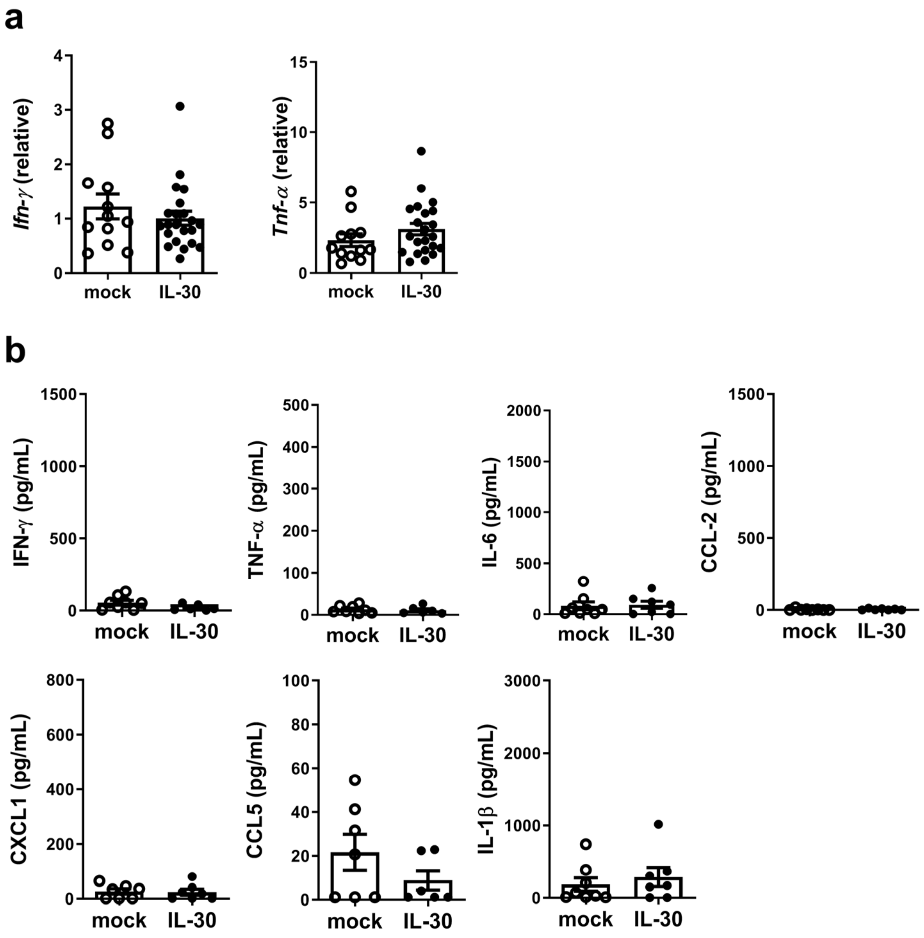

Figure S3. The administration of AAV-mIL-30 did not alter cytokines and chemokines in 2-OA-OVA-induced autoimmune cholangitis mice. Foxp3^{GFP} mice were injected with AAV-mIL-30 or AAV mock at 3 weeks after the first 2-OA-OVA immunization and sacrificed at Week 5. (a) Liver IFN-γ and TNF-α mRNA expression was detected using RT-qPCR. Relative quantification was performed by $2^{-\Delta CT}$ method and multiplied by 10000. (b) Serum levels of cytokines and chemokines were measured by performing fluorescent bead-based multiplex immunoassay. Each dot represents an individual mouse. n = 7–20 mice per group. All error bars denote ±SEM.
